# Finite Element Analysis and Biomechanical Testing to Analyze Fracture Displacement of Alveolar Ridge Splitting

**DOI:** 10.1155/2018/3579654

**Published:** 2018-10-14

**Authors:** Andres Stricker, Daniel Widmer, Boyko Gueorguiev, Dieter Wahl, Peter Varga, Fabian Duttenhoefer

**Affiliations:** ^1^University Hospital of Freiburg, Germany; ^2^AO Research Institute Davos, Switzerland

## Abstract

The alveolar ridge splitting technique enables reconstruction of atrophied alveolar ridges prior implantation. However, in cases of severe atrophy, there is an unpredictable risk of fracturing the buccal lamella during the expansion. Currently, there is no preoperative assessment to predict the maximum distraction of the lamella. The aim of this study was to develop a biomechanical model to mimic the alveolar ridge splitting and a finite element (FE) model to predict the experimental results. The biomechanical testing was conducted on porcine mandibles. To build the FE model high resolution peripheral quantitative computer tomography scans of one specimen was performed after the osteotomy outline, but before the lamella displacement. A servo-electric testing machine was used for the axial tension test to split the lamellae. Results showed, in line with clinical observations, that the lamellae broke primarily at the base of the splits with a median displacement of 1.27 mm. The FE model could predict fracture force and fracture displacement. Fracture force showed a nonlinear correlation with the height of the bone lamella. In conclusion, good correspondence between mechanical testing and virtual FE analysis showed a clinically relevant approach that may help to predict maximum lamella displacement to prevent fractures in the future.

## 1. Introduction

Implant-based dental rehabilitation of the severely atrophied alveolar ridge requires advanced augmentation techniques prior to implant placement. Besides classical onlay bone grafting and guided bone regeneration, the alveolar ridge splitting and expansion technique aims to enlarge the width of the alveolar ridge and thus provide a sufficient implantation site ([1–3]; Bassetti et al. 2016).

In comparison to onlay bone grafts the ridge splitting technique offers similar success rates when vertically sufficient but horizontally insufficient alveolar ridges are reconstructed. Moreover, this method avoids a second surgery site and decreases treatment time due to simultaneous implant placement (Altiparmak et al. 2017). Following longitudinal cutting of the ridge and preparation of two vertical relief incisions, the enlarged alveolar width is created by distraction of the outlined buccal segment and subsequent implant placement [4, 5]. Still, in cases of advanced atrophy, the success of the procedure is endangered by the high risk of fracturing the severely resorbed and thus fragile buccal lamella during expansion (Scipioni et al. 1994, [6–12]). This risk may be alleviated by preoperative prediction of the maximum possible magnitude of distraction prior to fracture. At this finite element analysis (FEA) may provide the adequate instrument to analyse the ridge splitting technique.

To date, finite element analysis is a common tool to virtually analyze mechanical strength as well as stress shielding areas in mechanics and in biomechanics. The application of this technique in the medical field is constantly increasing. Recent studies highlighted the potential of FEA analysis in dental medicine to evaluate biomechanical processes, especially in dental implantology and prosthodontic rehabilitation. FE based analysis of prosthetic dental crowns components, their physical and chemical properties [13], and the testing of prosthesis retention systems [14] provided valuable insight into patient specific dental rehabilitation. In a recent study, the FE-analysis of a three-dimensional model regarding the alteration of mechanical and prosthodontic components of dental implants gave insight into the highly debated discrepancy between implant survival and clinical success [15]. Moreover, FE-analysis allows the evaluation of biomechanical stress distribution in the stomatognathic system [16] and may provide information simulating the outcome of destructive tests [17].

However, up to date, there is no scientific insight with regard to the existing fracture mechanisms and the possibilities of fracture prediction. Therefore, the aim of this study was to develop a biomechanical model, implementing the bone splitting technique, together with a numerical model for prediction of the results from the intervention in order to simulate the surgical procedure and fracture behavior.

## 2. Materials and Methods

### 2.1. Experimental Model

Five porcine specimens (Sus scrofa f. domestica) aged between six and nine months were collected from the slaughterhouse. The full mandibles and maxillae were cut in half (sagittal) and stripped off soft tissue and periosteum to expose the bone on the edentulous part of the ridge. A total of five mandibles and one maxilla were enrolled in the experiment.

### 2.2. Surgical Procedure

**Figure 1:**
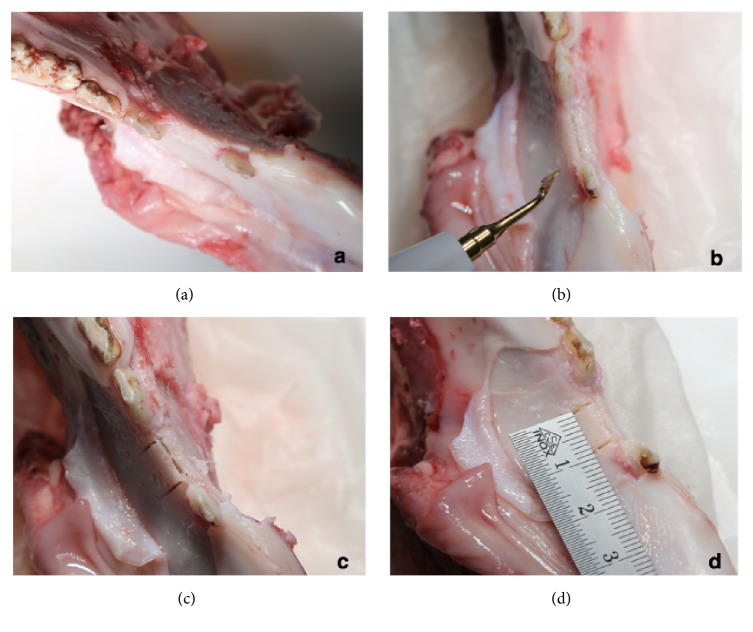
Osteotomy outline comprising a crestal cut and two buccal release cuts with an oscillating piezo saw.

Surgery was performed with an oscillating piezo saw using a blade thickness of 0.55 mm (Piezosurgery®, Mectron s.p.a., Carasco, Italy). The osteotomy outline comprised a crestal cut between 7 and 10 millimeters in the mesio-distal direction with four to eight millimeters depth, followed by two buccal release cuts of approximately eight millimeters on the mesial and distal end of the ridge osteotomy. The accordingly created solely apically pedicled buccal bone plate with a thickness between one and three millimeters was ready to get displaced in the outward direction for further investigation (Figure 1). During the preparation of the specimens the maxillae showed unsuitable anatomical dimension to simulate the splitting technique; hence only one maxilla specimen was prepared as comparison.

### 2.3. CT Scanning, Image Processing, and Finite Element Modelling

**Figure 2:**
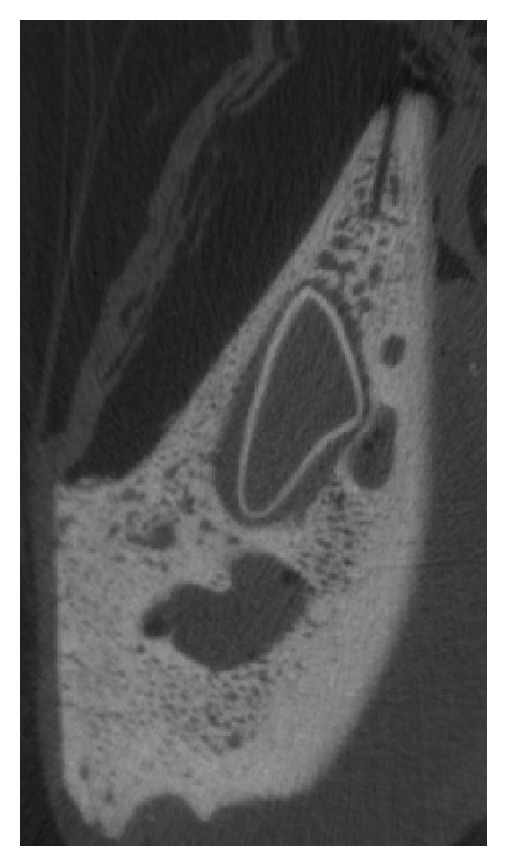
Acquisition of the split lamella by high resolution peripheral quantitative computer tomography (HR-pQCT).

High resolution peripheral quantitative computer tomography (HR-pQCT; XtremeCT, Scanco Medical AG, Brütisellen, Switzerland) scan of one specimen was performed after surgery but before testing. Scanning settings were 60 kVp voltage, 900 *μ*A current, and 82 *μ*m isotropic voxel size. The image grayscales were converted to bone mineral density (BMD, in mgHA/cm^3^) units using the in-built calibration curve of the scanner (Figure 2).

The pre-test HR-pQCT image was processed using ScanIP software (v7.0, Simpleware, Synopsys Inc., Mountain View, California, U.S.) to build a finite element (FE) model. The image was cropped to the region of interest around the selected split lamella. The bone domain of the sample was defined using a combination of global thresholding, manual segmentation, and fill, opening, and closing operations. The resulting image mask defined the outer contour of the split sample.

The domain of the sample mask was meshed with linear tetrahedral elements of element edge length of approximately 0.3mm around the cut and the bone flap, and approximately 1 mm at the other regions of the model. The conversion from HR-pQCT-based BMD to Young's modulus (E) was done with literature-based conversion rules [18]:

 
*E*_[Gpa]_ = 6.85 · *ρ*^1.49^ for trabecular bone [19]
 
*E*_[Gpa]_ = 10.5 · *ρ*^2.29^ for cortical bone [20]

**Figure 3:**
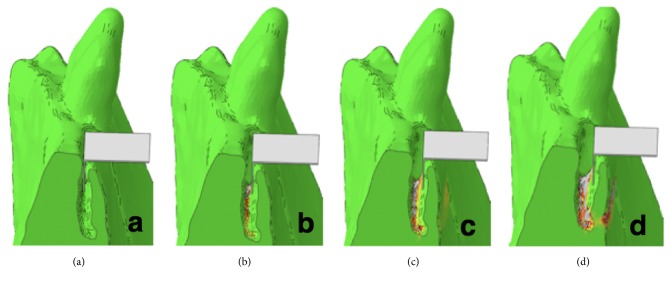
Fracture simulation of the split lamella based on the segmented test HR-pQCT images.

**Figure 4:**
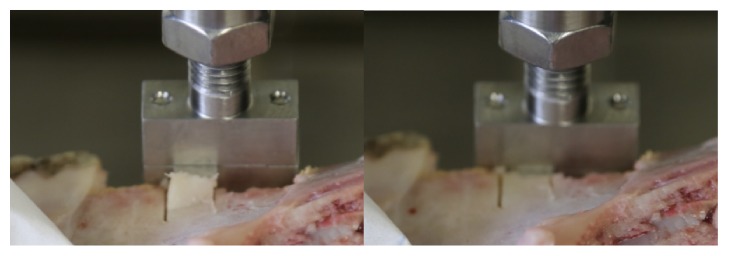
Biomechanical testing with a servo-electric material testing machine demonstrating fracture of the split lamella on the right side.

Plasticity was defined by implementing a custom hardening function. The mesh and material properties were exported and processed further in Abaqus CAE v6.12 (Dassault Systemes Simulia Corp., Providence, RI, USA). Displacement of the nodes located on the two sides of the sample was constrained in all three directions. A rigid plate was added to the model and aligned with the split in accordance with the experimental conditions. Frictionless contact condition was defined between the inner surface of the split and the plate. The displacement of this plate was prescribed in the direction normal to the split plane and constrained in the other two directions (Figure 3). The simulation was performed in Abaqus. The reaction force of the plate was computed for each analysis step and plotted against the plate displacement. Failure was defined based on the peak of this curve (Figure 4).

### 2.4. Biomechanical Testing

The distraction procedure was mimicked by a controlled opening of the buccal lamella. A material testing system (Instron 5866, Instron, Norwood, USA) equipped with a 1 kN load cell was used for tension test of the split lamellae. A stainless steel plate of 1 mm thickness was mounted between two metal blocks and fixed to the machine actuator perpendicular to the texting axis, i.e., horizontally. The embedded sample was aligned to match the split plane with the plane of the metal plate, i.e., horizontally, and mounted to the testing frame by means of a metal clamp (Figure 4). The plate was then moved in vertical direction by a quasi-static displacement at a rate of 5 mm/min. The motion of the crosshead was recorded and the force was measured with the load cell at 10 kHz. The test was stopped at contact loss between the stainless steel plate and the fractured lamella. The samples were kept wet during the test by spraying with PBS. Fracture load was defined as the peak of the force-displacement curve.

## 3. Results

The biomechanical tests resulted in a clinically relevant fracture mode where the lamellae broke primarily at the base of the splits. The displacement of the five mobilized buccal lamellae demonstrated similarity to clinical practice. Lamella dimensions at the coronal level had an average width of 8.2 mm (range 7.5 – 10.6 mm) and at bottom level of 8.8 mm (range 7.4 – 10.8 mm).

The average height relief in total was 7.0 mm consisting of side at left osteotomy of 7.0 mm (range 6.2 – 8.4 mm) and of 7.0 mm (6.2 – 8.3 mm) at right side. Experimental fracture forces were between 2.75 and 96.06 N (median 37.44 N) and displacements were between 0.62 and 2.93 mm (median 1.27 mm), respectively (Table 1). The fracture force showed a nonlinear correlation with the height of the bone lamella (Figure 6). No other significant correlations were found between experimental results and split geometries.

**Figure 5:**
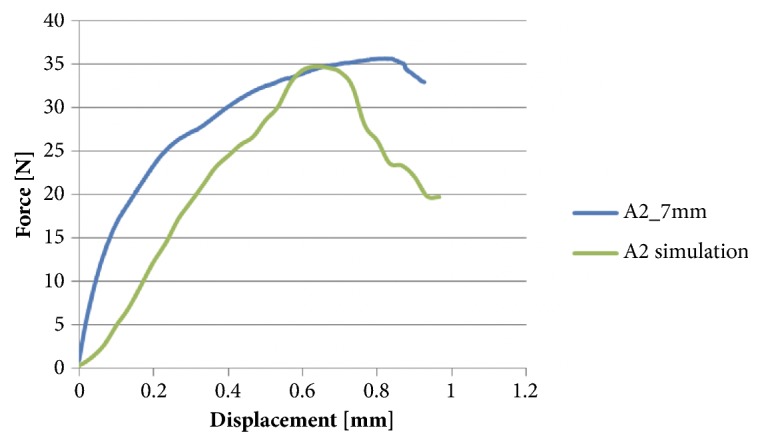
Experimental fracture force and displacement predicted by the FE model.

**Figure 6:**
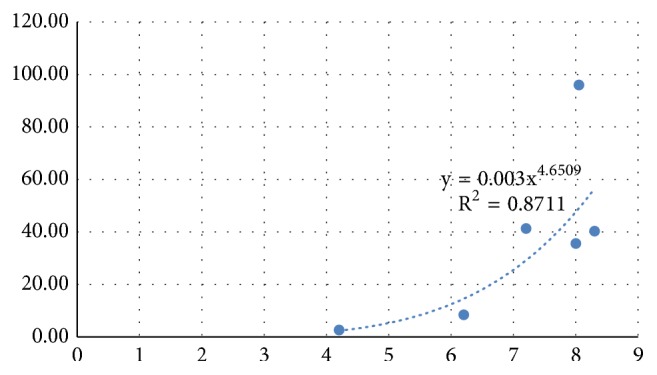
Experimental fracture force (N) and Average Height Relief (mm).

The FE model well predicted fracture force and fracture displacement. The yielded regions in the model exhibited good qualitative match with the fracture patterns (Figure 5).

## 4. Discussion

Fracture of the buccal lamella is one of the most common complications during the surgical intervention of alveolar ridge splitting [21]. To estimate the risk of malfracture in advance is of paramount interest to adapt the applied surgical protocol according to the maximal possible displacement. To date, there is no scientific approach to experimentally and numerically simulate the alveolar ridge splitting technique and gain insight into extension behavior of the buccal lamella and eventually its fracture mechanisms. However, computational tools such as FE analysis may provide a deeper insight. FE analysis is a common tool being introduced to reflect mechanical strength virtually and to analyze stress shielding areas in mechanics and in biomechanics.

In the current study, for the first time to the best of the authors' knowledge, FE simulation is used to analyze biomechanical behavior of a surgical technique related to an augmentation procedure enabling subsequent implant placement. Interestingly it is highly debated whether or not to design the model based on biologically parameters [22] or designing a numerical model per se [23]. Still, there is evidence in the literature that suggests to base the model entirely on biological parameters (Mellal et al. 2003). To evaluate this proof of principle, a pig model was considered appropriate for bone research because of its close similarity to human bone in terms of structure and bone mineral density [24, 25]. The FE model of a simple sample provided good prediction of fracture load and location. Further studies are required to demonstrate the capabilities of the FE model on a larger sample set. Moreover, the good bone quality of the used porcine model may not well represent the brittle human bone in advanced atrophy condition. Future studies should investigate lamellar fracture in more realistic samples. Our results showed that the fracture force correlated with the height of the bone lamella. It was shown previously that increasing the angle of load application is a key factor related to higher stress and strain level in the surrounding bone [26, 27]. This may be seen as a contradiction, since a larger lamellar height would correspond to a larger moment arm of the force and finally a larger bending moment acting at the base of the lamella. However, as it can be observed in Figure 2, the lamellar cross section was not uniform along the height, but increasing towards the base. Thus, the reason for the larger fracture load observed for higher lamellae may be the larger thickness at the base. However, the clinically more relevant fracture displacement was not correlated with the assessed lamellar geometries. It could be assumed that there is a difference of fracture behavior related to the thickness of the buccal lamella to be displaced, but lamellar thickness was not measured for all samples. Moreover it can be assumed that the cancellous part of the bone accounts for the possible maximum level of displacement. It was suggested in previous investigations to take the nonlinear elasticity and transverse isotropy of bone into account when creating numerical models [28]. This assumption was based on the fact that the often varying and inhomogeneous composition of materials leads to anisotropy and hence difficulties when calculating the elastic constant measurement [29]. It is known from orthopedic implant placement in total knee arthroplasty that the plastic deformation in the trabecular bone is highly dependent on the plasticity formulation implemented [30].

To get further insight into fracture mechanisms this study advocates that future experiments may focus on the lamellar thickness at the bottom of the split as there is still no good predictor for fracture displacement. Nevertheless, our FE models incorporate the aspects of lamellar geometry, material property distribution, and loading mode. These models are therefore expected to better predict lamellar fracture compared to simple geometrical parameters. This question should be investigated in future studies.

## 5. Conclusion

We conclude that the conducted FE modeling presents a novel approach to better understand the biomechanics of the alveolar ridge splitting technique. However, further studies, applying the human anatomy and its tissue response, are necessary to analyze more patient-specific conditions in a larger sample set. It has to be deciphered how image resolution and quality of the clinically available image acquisition systems affect the level of the FE-based predictions. Still, the good correspondence between mechanical testing and virtual FE analysis may be a first step to predict the maximum level of lamella displacement and hence prevent fractures of the split lamellae.

## Figures and Tables

**Table 1:** Dimensions of osteotomies of the split lamellae and results of the biomechanical testing.

Sample		Split geometry						Experimental results	
ID	Anatomical location	Height Relief Left mm	Height Relief Right mm	Average Height Relief mm	Width at Bottom mm	Width at Top mm	Average Width mm	Max Load N	Extension at Max Load mm
A1	Mandibula	8,3	7,8	8,05	10,8	10,6	10,7	96,07	1,42
A2	Mandibula	7,7	8,3	8	7,4	7,5	7,45	35,63	0,83
A3	Mandibula	7,1	7,2	7,2	8,1	8,1	8,1	41,36	0,62
A4	Mandibula	8,3	8,3	8,3	9,2	9,3	9,25	40,37	2,94
A5	Mandibula	6,2	6,2	6,2	8,1	8,1	8,1	8,48	1,09
A5	Maxilla	4,2	4,2	4,2	9,3	9,3	9,3	2,75	0,71

Mean				7,0			8,8	37,4	1,3
Standard deviation				1,6			1,2	33,2	0,9

## Data Availability

The data of the submitted paper is available upon e-mail request to the corresponding author.
